# Supplementary material for: A Likelihood Approach for Real-Time Calibration of Stochastic Compartmental Epidemic Models
Source: PLoS Comput Biol. 2017 Jan 17;13(1):e1005257. doi: 10.1371/journal.pcbi.1005257 (PMC5240920; doi:10.1371/journal.pcbi.1005257)
Supplement: S1 File — (TAR.GZ) [file pcbi.1005257.s014.tar.gz › HSPH_Online-SI-Revision/Additions/MSS11/n100-extreme-SEITR/n100-extreme-SEITR_NEW_table8.pdf]

1	R0	{1.38654, 9.85565, 1.}	{2.6365, 15.4074, 0.}	{{2.6365, 15.4074, 0.}, {2.6365, 15.4074, 0.}}	{90.1495, 56.3303, 100.}	inf	658.	1037.22	{1024.09, 1048.94}	57.6324	0.197623	newly	382.	393.66	{372., 413.}	4.11518	0.0174297	Reff	0.108483	0.198338	{0.197944, 0.198677}	82.8288	ar	92379.	92723.3	{92695., 92756.}	0.372725
2	R0	{2.36629, 12.2029, 0.1}	{2.95668, 7.22215, 1.}	{{2.95668, 7.22215, 1.}, {2.95668, 7.22215, 1.}}	{24.95, 40.8161, 900.}	inf	689.	1.16026	{1.02074, 1.29994}	99.8316	2.77573	newly	300.	0.58	{0., 1.}	99.8067	2.89712	Reff	0.0235682	0.0499336	{0.0499291, 0.0499377}	111.868	ar	99022.	98311.	{98311., 98311.}	0.717972
3	R0	{2.32253, 12.836, 1.}	{2.97612, 8.62388, 4.}	{{2.97612, 8.62388, 4.}, {2.97612, 8.62388, 4.}}	{28.1413, 32.8149, 300.}	inf	20.	1.17372	{1.02935, 1.38236}	99.8896	2.9598	newly	463.	0.51	{0., 1.}	99.8898	3.15558	Reff	0.0245491	0.063654	{0.0636471, 0.0636581}	159.292	ar	98997.	97861.1	{97861., 97861.}	1.14741
4	R0	{1.79075, 11.2058, 1.}	{2.94803, 11.5745, 4.}	{{2.94803, 11.5745, 4.}, {2.94803, 11.5745, 4.}}	{64.6254, 3.2903, 300.}	inf	31.	93.3274	{85.3925, 101.559}	88.7286	0.948899	newly	412.	43.32	{36., 50.}	89.4854	0.981667	Reff	0.0574114	0.117086	{0.116834, 0.117314}	103.942	ar	96920.	96040.1	{96031., 96050.}	0.907883
5	R0	{2.26952, 9.62371, 0.1}	{2.88144, 1.1121, 1.}	{{2.88144, 1.1121, 1.}, {2.88144, 1.1121, 1.}}	{26.9624, 88.4442, 900.}	inf	0.	1.16689	{1.02964, 1.32963}	99.376	2.20674	newly	78.	1.04	{1., 1.}	98.6667	1.88005	Reff	0.0257137	0.0380014	{0.0379966, 0.0380052}	47.7866	ar	98881.	98681.	{98681., 98681.}	0.202223
6	R0	{1.57191, 6.89004, 0.1}	{2.95668, 7.22215, 1.}	{{2.95668, 7.22215, 1.}, {2.95668, 7.22215, 1.}}	{88.0947, 4.82017, 900.}	inf	5.	1.27479	{1.04864, 1.54304}	97.6393	1.63249	newly	40.	0.67	{0., 1.}	98.325	1.97525	Reff	0.077951	0.148329	{0.14832, 0.148335}	90.2842	ar	95046.	94983.3	{94983., 94984.}	0.065926
7	R0	{1.99226, 17.2339, 1.}	{2.96797, 18.4042, 0.}	{{2.96797, 18.4042, 0.}, {2.96797, 18.4042, 0.}}	{48.9752, 6.79072, 100.}	inf	78.	69.2773	{59.5859, 80.4873}	97.5819	1.61986	newly	982.	22.05	{16., 27.}	97.7546	1.65692	Reff	0.0456427	0.153288	{0.152951, 0.153569}	235.843	ar	97992.	94847.4	{94835., 94861.}	3.20901
8	R0	{2.63369, 15.1879, 0.1}	{2.99949, 5.42517, 0.}	{{2.99949, 5.42517, 0.}, {2.99949, 5.42517, 0.}}	{13.8894, 64.2797, 100.}	inf	5.	3.0822	{1.2257, 5.25042}	99.7842	2.72419	newly	510.	2.27	{1., 4.}	99.5549	2.46856	Reff	0.0154861	0.0605273	{0.060457, 0.0605827}	290.849	ar	99451.	97982.2	{97980., 97985.}	1.47689
9	R0	{2.53271, 9.90552, 0.1}	{2.99949, 5.42517, 0.}	{{2.99949, 5.42517, 0.}, {2.99949, 5.42517, 0.}}	{18.4302, 45.2309, 100.}	inf	2.	1.09897	{1.01743, 1.20146}	99.3573	2.19302	newly	96.	0.71	{0., 1.}	99.2604	2.27227	Reff	0.0167159	0.0249528	{0.0249497, 0.0249553}	49.2761	ar	99355.	99168.	{99168., 99168.}	0.188174
10	R0	{2.48791, 18.9003, 2.}	{2.91192, 15.1048, 3.}	{{2.91192, 15.1048, 3.}, {2.91192, 15.1048, 3.}}	{17.043, 20.0815, 50.}	inf	42.	558.539	{543.573, 576.826}	85.1492	0.828348	newly	1167.	211.	{196., 230.}	81.9195	0.743672	Reff	0.0212716	0.119373	{0.118836, 0.119779}	461.185	ar	99291.	95976.1	{95954., 95999.}	3.33859
11	R0	{2.84414, 18.4765, 1.}	{2.9884, 12.8326, 0.}	{{2.9884, 12.8326, 0.}, {2.9884, 12.8326, 0.}}	{5.07205, 30.5464, 100.}	inf	13.	7.75133	{2.25083, 13.797}	99.7532	2.70026	newly	1003.	3.22	{1., 7.}	99.679	2.67154	Reff	0.0117463	0.106364	{0.106183, 0.106529}	805.515	ar	99654.	96441.7	{96435., 96448.}	3.22343
12	R0	{2.29369, 14.622, 0.1}	{2.99092, 10.3743, 0.}	{{2.99092, 10.3743, 0.}, {2.99092, 10.3743, 0.}}	{30.3976, 29.05, 100.}	inf	14.	3.32152	{1.46478, 5.85897}	99.7567	2.67342	newly	542.	1.6	{0., 3.}	99.7048	2.74309	Reff	0.0247948	0.0734772	{0.0733876, 0.0735325}	196.341	ar	98982.	97543.6	{97541., 97547.}	1.45315
13	R0	{2.73413, 16.7529, 1.}	{2.98388, 9.60029, 3.}	{{2.98388, 9.60029, 3.}, {2.98388, 9.60029, 3.}}	{9.13455, 42.6948, 200.}	inf	10.	6.00222	{1.64641, 9.22417}	99.7072	2.60113	newly	706.	3.05	{0., 6.}	99.568	2.55186	Reff	0.0138347	0.0763873	{0.0762777, 0.0765117}	452.143	ar	99543.	97440.6	{97436., 97445.}	2.1121
14	R0	{1.79554, 16.5943, 2.}	{2.93978, 19.9817, 0.}	{{2.93978, 19.9817, 0.}, {2.93978, 19.9817, 0.}}	{63.7265, 20.4132, 100.}	inf	185.	1296.19	{1278.27, 1314.25}	65.6821	0.464504	newly	1313.	391.67	{370., 416.}	70.1698	0.525726	Reff	0.0662734	0.186876	{0.186342, 0.187396}	181.978	ar	96821.	93930.7	{93899., 93962.}	2.98522
15	R0	{1.91429, 13.7771, 1.}	{2.96797, 18.4042, 0.}	{{2.96797, 18.4042, 0.}, {2.96797, 18.4042, 0.}}	{55.043, 33.5855, 100.}	inf	37.	5.34439	{1.87419, 9.23074}	99.6362	2.51851	newly	621.	1.71	{0., 4.}	99.7246	2.81304	Reff	0.0501544	0.1223	{0.122178, 0.122403}	143.847	ar	97541.	95880.2	{95876., 95885.}	1.70269
16	R0	{1.42094, 10.5871, 2.}	{2.85582, 17.2484, 2.}	{{2.85582, 17.2484, 2.}, {2.85582, 17.2484, 2.}}	{100.981, 62.9193, 0.}	inf	126.	1203.88	{1187.78, 1218.91}	0.876284	0.00379676	newly	644.	416.38	{393., 441.}	35.3447	0.189792	Reff	0.103388	0.211306	{0.210872, 0.211762}	104.382	ar	93044.	92920.3	{92879., 92951.}	0.132959
17	R0	{1.45515, 6.16466, 0.1}	{2.9751, 8.95585, 1.}	{{2.9751, 8.95585, 1.}, {2.9751, 8.95585, 1.}}	{104.453, 45.2773, 900.}	inf	5.	422.15	{411.384, 432.937}	744.3	0.926417	newly	41.	244.57	{227., 260.}	496.512	0.775102	Reff	0.0982954	0.190045	{0.189708, 0.190355}	93.3404	ar	93254.	93709.8	{93688., 93732.}	0.488719
18	R0	{2.85669, 13.2416, 0.1}	{2.59046, 1.09671, 0.}	{{2.59046, 1.09671, 0.}, {2.59046, 1.09671, 0.}}	{9.31969, 91.7177, 100.}	inf	1.	1.15866	{1.01911, 1.31907}	99.8511	2.82899	newly	314.	1.04	{1., 1.}	99.6688	2.48489	Reff	0.0106269	0.0297861	{0.029782, 0.0297896}	180.29	ar	99644.	98850.	{98850., 98850.}	0.796797
19	R0	{2.56417, 17.9558, 0.1}	{2.86001, 13.5542, 0.}	{{2.86001, 13.5542, 0.}, {2.86001, 13.5542, 0.}}	{11.5373, 24.5134, 100.}	inf	35.	6.23276	{1.37522, 11.0924}	99.8054	2.80642	newly	1136.	2.52	{0., 5.}	99.7782	2.89173	Reff	0.01818	0.112706	{0.112561, 0.112843}	519.947	ar	99389.	96060.2	{96054., 96066.}	3.34926
20	R0	{2.27517, 11.1864, 0.1}	{2.95668, 7.22215, 1.}	{{2.95668, 7.22215, 1.}, {2.95668, 7.22215, 1.}}	{29.9542, 35.4381, 900.}	inf	9.	1.15051	{1.02971, 1.29247}	99.7499	2.60346	newly	219.	0.58	{0., 1.}	99.7352	2.76044	Reff	0.0255502	0.0470363	{0.0470321, 0.0470397}	84.0941	ar	98894.	98409.1	{98409., 98409.}	0.490363
21	R0	{2.35735, 10.4735, 0.1}	{2.99949, 5.42517, 0.}	{{2.99949, 5.42517, 0.}, {2.99949, 5.42517, 0.}}	{27.2401, 48.201, 100.}	inf	3.	1.15437	{1.02805, 1.29523}	99.6139	2.4151	newly	156.	0.74	{0., 1.}	99.5256	2.47409	Reff	0.0230549	0.0383589	{0.038354, 0.0383627}	66.3808	ar	99036.	98721.1	{98721., 98721.}	0.318016
22	R0	{1.79551, 11.9179, 2.}	{2.9884, 12.8326, 0.}	{{2.9884, 12.8326, 0.}, {2.9884, 12.8326, 0.}}	{66.4372, 7.67498, 100.}	inf	31.	59.145	{50.3023, 66.6519}	93.3245	1.17785	newly	400.	24.84	{19., 30.}	93.79	1.21395	Reff	0.0555172	0.118037	{0.117764, 0.118275}	112.614	ar	97033.	96058.2	{96047., 96068.}	1.00465
23	R0	{1.23545, 6.31687, 0.1}	{2.49869, 9.80052, 1.}	{{2.49869, 9.80052, 1.}, {2.49869, 9.80052, 1.}}	{102.249, 55.1483, 900.}	inf	19.	79.2584	{70.6024, 87.0074}	19.941	0.0981159	newly	79.	44.32	{37., 52.}	43.8987	0.254885	Reff	0.139655	0.28342	{0.283222, 0.283632}	102.942	ar	88739.	88689.	{88672., 88706.}	0.0562999
24	R0	{2.24089, 10.1789, 0.1}	{2.95668, 7.22215, 1.}	{{2.95668, 7.22215, 1.}, {2.95668, 7.22215, 1.}}	{31.9422, 29.0478, 900.}	inf	7.	1.13913	{1.02583, 1.27554}	99.6717	2.48554	newly	171.	0.6	{0., 1.}	99.6491	2.63999	Reff	0.025703	0.0443461	{0.0443413, 0.0443494}	72.5327	ar	98868.	98500.1	{98500., 98500.}	0.372153
25	R0	{1.57298, 10.2607, 0.1}	{2.86001, 13.5542, 0.}	{{2.86001, 13.5542, 0.}, {2.86001, 13.5542, 0.}}	{81.821, 32.0985, 100.}	inf	47.	28.9988	{18.3491, 39.2519}	96.6668	1.49428	newly	443.	12.24	{7., 18.}	97.237	1.58575	Reff	0.0819837	0.17446	{0.174167, 0.174765}	112.799	ar	94957.	93906.4	{93893., 93918.}	1.10643
26	R0	{1.2154, 8.2576, 0.1}	{2.57756, 15.2661, 2.}	{{2.57756, 15.2661, 2.}, {2.57756, 15.2661, 2.}}	{112.075, 84.8736, 1900.}	inf	88.	2346.62	{2328.73, 2364.66}	346.976	0.650275	newly	345.	910.53	{881., 941.}	163.922	0.42135	Reff	0.143952	0.260601	{0.260089, 0.261061}	81.0335	ar	88421.	90679.	{90626., 90733.}	2.55367
27	R0	{2.22841, 18.7983, 2.}	{2.99088, 18.9406, 2.}	{{2.99088, 18.9406, 2.}, {2.99088, 18.9406, 2.}}	{34.2161, 0.756936, 0.}	inf	67.	248.609	{237.66, 258.12}	92.8725	1.1473	newly	1079.	78.76	{68., 89.}	92.7006	1.13914	Reff	0.0322005	0.142109	{0.141797, 0.142429}	341.324	ar	98756.	95289.4	{95274., 95304.}	3.5103
28	R0	{1.27778, 5.36767, 1.}	{2.55086, 6.16955, 1.}	{{2.55086, 6.16955, 1.}, {2.55086, 6.16955, 1.}}	{99.632, 14.939, 0.}	inf	7.	101.431	{92.4546, 109.616}	198.328	0.473626	newly	32.	77.12	{68., 87.}	141.	0.380049	Reff	0.130065	0.25811	{0.257901, 0.258332}	98.4468	ar	89843.	89917.7	{89901., 89934.}	0.0831228
29	R0	{2.38042, 15.8901, 2.}	{2.99519, 12.6436, 3.}	{{2.99519, 12.6436, 3.}, {2.99519, 12.6436, 3.}}	{25.8259, 20.4307, 50.}	inf	37.	101.682	{91.5255, 111.391}	95.6266	1.36053	newly	849.	44.32	{36., 52.}	94.7797	1.28688	Reff	0.0237804	0.0976226	{0.0973198, 0.0979236}	310.517	ar	99112.	96751.1	{96739., 96764.}	2.38207
30	R0	{2.53188, 12.7607, 0.1}	{2.77569, 1.01414, 2.}	{{2.77569, 1.01414, 2.}, {2.77569, 1.01414, 2.}}	{9.62952, 92.0527, 1900.}	inf	11.	1.39215	{1.05816, 1.82524}	99.8612	2.86786	newly	428.	1.33	{1., 2.}	99.6893	2.53835	Reff	0.0182295	0.0480918	{0.0480795, 0.048101}	163.812	ar	99317.	98267.3	{98267., 98268.}	1.05689
31	R0	{1.24482, 10.5471, 1.}	{2.29055, 13.7413, 0.}	{{2.29055, 13.7413, 0.}, {2.29055, 13.7413, 0.}}	{84.0066, 30.2852, 100.}	inf	170.	967.26	{948.134, 983.609}	24.4328	0.121719	newly	689.	411.44	{392., 432.}	40.2845	0.224258	Reff	0.142009	0.272363	{0.271967, 0.272792}	91.793	ar	89097.	88466.3	{88425., 88511.}	0.707835
32	R0	{2.30446, 17.9607, 1.}	{2.91192, 15.1048, 3.}	{{2.91192, 15.1048, 3.}, {2.91192, 15.1048, 3.}}	{26.3604, 15.9007, 200.}	inf	48.	7.93263	{2.19413, 15.5158}	99.7641	2.73619	newly	1094.	2.89	{0., 6.}	99.7358	2.78037	Reff	0.0266626	0.132786	{0.132535, 0.132952}	398.022	ar	99028.	95441.4	{95434., 95450.}	3.62182
33	R0	{2.31579, 11.8236, 2.}	{2.97612, 8.62388, 4.}	{{2.97612, 8.62388, 4.}, {2.97612, 8.62388, 4.}}	{28.5142, 27.0622, 100.}	inf	5.	1.15554	{1.02974, 1.30388}	99.7577	2.61749	newly	197.	0.51	{0., 1.}	99.7411	2.78447	Reff	0.0234821	0.0444884	{0.0444836, 0.0444921}	89.4564	ar	99012.	98505.1	{98505., 98505.}	0.511999
34	R0	{1.6678, 10.6744, 1.}	{2.9884, 12.8326, 0.}	{{2.9884, 12.8326, 0.}, {2.9884, 12.8326, 0.}}	{79.1819, 20.2184, 100.}	inf	36.	366.013	{355.408, 376.08}	36.0118	0.193994	newly	278.	157.42	{145., 170.}	43.3741	0.247795	Reff	0.0712985	0.134985	{0.134681, 0.135283}	89.3246	ar	95809.	95538.3	{95527., 95551.}	0.282552
35	R0	{1.65476, 7.07559, 0.1}	{2.99949, 5.42517, 0.}	{{2.99949, 5.42517, 0.}, {2.99949, 5.42517, 0.}}	{81.2646, 23.3256, 100.}	inf	5.	1.26743	{1.03199, 1.58195}	98.1631	1.74151	newly	50.	0.87	{0., 2.}	98.26	1.91984	Reff	0.0705259	0.13002	{0.13001, 0.130027}	84.358	ar	95746.	95665.3	{95665., 95666.}	0.0843168
36	R0	{2.71807, 18.5959, 0.1}	{2.9884, 12.8326, 0.}	{{2.9884, 12.8326, 0.}, {2.9884, 12.8326, 0.}}	{9.94552, 30.9923, 100.}	inf	24.	9.05679	{2.17186, 16.1821}	99.7601	2.7218	newly	1211.	3.89	{0., 8.}	99.6788	2.71544	Reff	0.0154386	0.130262	{0.130023, 0.130459}	743.743	ar	99540.	95642.4	{95634., 95651.}	3.91563
37	R0	{2.6202, 12.7552, 0.1}	{2.96307, 14.3219, 5.}	{{2.96307, 14.3219, 5.}, {2.96307, 14.3219, 5.}}	{13.0858, 12.2827, 4900.}	inf	9.	1.12563	{1.02016, 1.26258}	99.8711	2.89102	newly	383.	0.36	{0., 1.}	99.906	3.2232	Reff	0.016088	0.0442942	{0.04429, 0.0442973}	175.324	ar	99426.	98505.1	{98505., 98505.}	0.926267
38	R0	{1.47672, 9.81651, 1.}	{2.73634, 13.3345, 1.}	{{2.73634, 13.3345, 1.}, {2.73634, 13.3345, 1.}}	{85.2986, 35.8372, 0.}	inf	40.	495.658	{484.568, 506.682}	12.8897	0.0599971	newly	323.	210.01	{193., 227.}	34.9814	0.18779	Reff	0.0934468	0.176257	{0.175949, 0.176539}	88.6175	ar	93815.	93662.6	{93641., 93682.}	0.16249
39	R0	{1.90004, 12.3313, 1.}	{2.91946, 11.8546, 2.}	{{2.91946, 11.8546, 2.}, {2.91946, 11.8546, 2.}}	{53.6523, 3.86561, 100.}	inf	30.	322.647	{313.092, 332.53}	69.5328	0.516284	newly	464.	146.37	{133., 158.}	68.4547	0.50203	Reff	0.0517191	0.101841	{0.101539, 0.102114}	96.9116	ar	97413.	96547.9	{96533., 96561.}	0.888105
40	R0	{2.64218, 18.5237, 2.}	{2.99519, 12.6436, 3.}	{{2.99519, 12.6436, 3.}, {2.99519, 12.6436, 3.}}	{13.3604, 31.7434, 50.}	inf	25.	162.592	{153.272, 171.653}	94.1472	1.23305	newly	874.	69.94	{61., 80.}	91.9977	1.09897	Reff	0.0162494	0.0975055	{0.0972271, 0.0977805}	500.056	ar	99474.	96762.6	{96750., 96774.}	2.72575
41	R0	{1.6945, 7.17996, 0.1}	{2.99949, 5.42517, 0.}	{{2.99949, 5.42517, 0.}, {2.99949, 5.42517, 0.}}	{77.0135, 24.4401, 100.}	inf	4.	1.28947	{1.05042, 1.59615}	98.7481	1.90833	newly	74.	0.85	{0., 1.}	98.8514	2.08913	Reff	0.0626626	0.114092	{0.114083, 0.114099}	82.0736	ar	96332.	96196.4	{96196., 96197.}	0.140815
42	R0	{1.84559, 18.0074, 1.}	{2.96797, 18.4042, 0.}	{{2.96797, 18.4042, 0.}, {2.96797, 18.4042, 0.}}	{60.8143, 2.20357, 100.}	inf	180.	3904.67	{3889.61, 3923.25}	1.71979	0.00753624	newly	1374.	1252.47	{1216., 1288.}	8.84498	0.0403263	Reff	0.0586713	0.101722	{0.101171, 0.102164}	73.3763	ar	97358.	96981.9	{96947., 97014.}	0.386265
43	R0	{2.24965, 17.0361, 2.}	{2.91192, 15.1048, 3.}	{{2.91192, 15.1048, 3.}, {2.91192, 15.1048, 3.}}	{29.439, 11.3363, 50.}	inf	59.	611.934	{597.802, 624.37}	80.4056	0.707923	newly	1018.	230.79	{213., 247.}	77.3291	0.645232	Reff	0.0319	0.116129	{0.115753, 0.116494}	264.042	ar	98770.	96091.8	{96068., 96112.}	2.71153
44	R0	{2.54324, 15.7967, 0.1}	{2.98388, 9.60029, 3.}	{{2.98388, 9.60029, 3.}, {2.98388, 9.60029, 3.}}	{17.3259, 39.226, 2900.}	inf	13.	4.56917	{1.39055, 8.36064}	99.7186	2.63188	newly	626.	2.24	{0., 5.}	99.6422	2.62757	Reff	0.0177772	0.0695671	{0.0694532, 0.0696588}	291.327	ar	99354.	97668.9	{97665., 97672.}	1.69606
45	R0	{2.54943, 14.8032, 0.1}	{2.99949, 5.42517, 0.}	{{2.99949, 5.42517, 0.}, {2.99949, 5.42517, 0.}}	{17.6535, 63.3514, 100.}	inf	8.	3.97288	{1.49981, 6.87377}	99.7087	2.60684	newly	536.	2.88	{1., 5.}	99.4627	2.37948	Reff	0.0170047	0.0610405	{0.0609507, 0.0611131}	258.963	ar	99380.	97965.2	{97962., 97968.}	1.42359
46	R0	{2.37755, 12.6461, 2.}	{2.99949, 5.42517, 0.}	{{2.99949, 5.42517, 0.}, {2.99949, 5.42517, 0.}}	{26.159, 57.1001, 100.}	inf	9.	1.18555	{1.02215, 1.38469}	99.8671	2.88	newly	377.	0.76	{0., 1.}	99.7984	2.8443	Reff	0.0224678	0.0553351	{0.0553288, 0.0553398}	146.286	ar	99096.	98155.1	{98155., 98155.}	0.949483
47	R0	{1.69279, 11.6636, 1.}	{2.71225, 12.7047, 1.}	{{2.71225, 12.7047, 1.}, {2.71225, 12.7047, 1.}}	{60.2235, 8.92615, 0.}	inf	61.	242.423	{232.18, 252.952}	79.3507	0.685312	newly	550.	104.58	{95., 115.}	80.9855	0.722082	Reff	0.0676608	0.13533	{0.135034, 0.135603}	100.012	ar	96214.	95048.9	{95033., 95065.}	1.21098
48	R0	{1.82402, 10.0038, 1.}	{2.9751, 8.95585, 1.}	{{2.9751, 8.95585, 1.}, {2.9751, 8.95585, 1.}}	{63.1065, 10.4755, 0.}	inf	27.	4.2309	{1.32349, 8.4198}	99.1418	2.14315	newly	248.	2.18	{1., 4.}	99.121	2.20431	Reff	0.054447	0.104151	{0.104015, 0.104228}	91.2892	ar	97076.	96499.7	{96496., 96504.}	0.5937
49	R0	{1.6424, 11.1667, 2.}	{2.99939, 13.7076, 1.}	{{2.99939, 13.7076, 1.}, {2.99939, 13.7076, 1.}}	{82.6226, 22.7545, 50.}	inf	29.	1773.3	{1760.24, 1788.33}	163.884	0.421406	newly	321.	724.69	{692., 750.}	125.76	0.353436	Reff	0.0752383	0.10524	{0.104778, 0.105584}	39.8751	ar	95546.	96692.6	{96668., 96712.}	1.2
50	R0	{2.05638, 14.3048, 1.}	{2.9884, 12.8326, 0.}	{{2.9884, 12.8326, 0.}, {2.9884, 12.8326, 0.}}	{45.3232, 10.2917, 100.}	inf	38.	5.68141	{2.06363, 10.4073}	99.6425	2.52735	newly	636.	2.55	{0., 5.}	99.5991	2.6178	Reff	0.0397498	0.106217	{0.106064, 0.10632}	167.214	ar	98194.	96446.5	{96442., 96452.}	1.77966
